# Nebulized tranexamic acid for hemoptysis in critically and non-critically ill patients: A retrospective analysis

**DOI:** 10.2478/jccm-2025-0031

**Published:** 2025-07-31

**Authors:** Nancy Bethuel, Chris Naum, Cynthia Brown

**Affiliations:** Indiana University, Purdue University Indianapolis, Indianapolis, Indiana, USA; Indiana University, Indianapolis, Indiana, USA; Indiana University School of Medicine, Indianapolis, Indiana, USA

**Keywords:** hemoptysis, tranexamic acid, respiratory failure, ICU, bronchoscopy

## Abstract

**Introduction:**

Hemoptysis is a commonly encountered diagnosis caused by blood originating from the respiratory tract. Current pharmacological guideline recommendations for treatment do not exist. Tranexamic acid is a synthetic anti-fibrinolytic used in the management of various bleeding complications. Tranexamic acid has gained popularity for the treatment of hemoptysis with limited side effect knowledge. Our aim is to describe the clinical characteristics of patients receiving nebulized tranexamic acid for hemoptysis and compare clinical outcomes to those of patients receiving supportive care.

**Materials and Methods:**

This is a retrospective descriptive analysis performed in medical and ICU units at three tertiary hospitals. All patients were hospitalized with hemoptysis between January 1st, 2018 – December 31st, 2021. Demographic information, severity variables, and clinical outcomes were collected from medical records. For statistical analysis, we used t-test for continuous variables, chi-square or fishers’ exact test for categorical variables, and propensity analysis to adjust for disease severity and underlying medical conditions.

**Results:**

488 patients were identified; 96 received tranexamic acid. There were slightly more smokers in the no TXA group (p = 0.04) but otherwise the two groups were similar in terms of demographic characteristics. The average length of hospital and ICU stay, need for mechanical ventilation or bronchoscopy, and mortality were significantly higher in the tranexamic acid group (p<0.01). The propensity analysis showed higher odds of death with nebulized tranexamic acid use, OR 2.51 (1.56–4.02).

**Conclusions:**

There appears to be an indication bias for tranexamic acid based on disease severity without an obvious improvement in clinical outcomes. Our analysis suggests that nebulized tranexamic acid for hemoptysis may be potentially harmful, and further larger prospective research is warranted.

## Introduction

Hemoptysis is caused by blood originating from the respiratory tract [1, 2]. It is a highly prevalent diagnosis with common etiologies including respiratory infections, bronchiectasis, and lung cancer. Almost 10% of patients presenting with hemoptysis will require intensive care unit (ICU) admission. The standard management approach is treatment of the underlying cause [1, 3–4] while providing appropriate supportive care. Without immediate intervention, it has been associated with a mortality as high as 38% in non-massive hemoptysis due to asphyxiation [5]. Current pharmacological guideline recommendations for treatment do not exist.

Tranexamic acid (TXA) is a synthetic anti-fibrinolytic agent used in the management of various bleeding complications [1,2,3]. It prevents fibrinolysis by binding lysine receptors to prevent the conversion of plasminogen to plasmin and inhibits the action of plasmin on fibrin [1, 2]. The argument for using TXA is that it causes an increase in anti-fibrinolytic activity at sites of bleeding and subsequently decreases or stops bleeding [3]. There is an extensive amount of evidence supporting the use of intravenous TXA to decrease bleeding complications in trauma and post-operatively [1, 3]. However, there are few studies looking at the use of TXA in the management of hemoptysis, and these show conflicting benefits [2, 4]. Various routes of administration (oral, topical, nebulized, and intravenous) and different drug dosages have been tested with inconsistent results [2–3, 5–6].

While TXA is considered to be a relatively benign therapy, there have been consistent reports of increased clotting risk and bronchospasm [2, 4]. At higher doses (>2 g), seizures have also been reported post-operatively with intravenous TXA use [6]. Most recently Hardin et al. [7] reported a case of neurotoxicity attributed to nebulized TXA. Nevertheless, nebulized TXA is being increasingly used to manage patients presenting with hemoptysis.

Thus, we performed a retrospective descriptive analysis of the effectiveness of nebulized TXA in the management of hemoptysis compared to supportive management. Our primary objective was to assess the difference in ICU and hospital length of stay of patients receiving nebulized TXA for hemoptysis compared to patients receiving supportive care alone. Secondary objectives compared in-hospital mortality, adverse drug effects and the need for invasive procedures including bronchoscopy and interventional radiology (IR) intervention.

## Materials and methods

We performed a retrospective chart review of patients hospitalized with hemoptysis between January 1^st^, 2018, and December 31^st^, 2021. This study was conducted in accordance with the amended Declaration of Helsinki. The Indiana University institutional review board approved the protocol (IRB #16704) on September 29^th^, 2022. The protocol was titled “Our experience with nebulized tranexamic acid for hemoptysis in critical and non-critically ill patients: A retrospective analysis”. Informed written consent was not required.

**Fig. 1.**
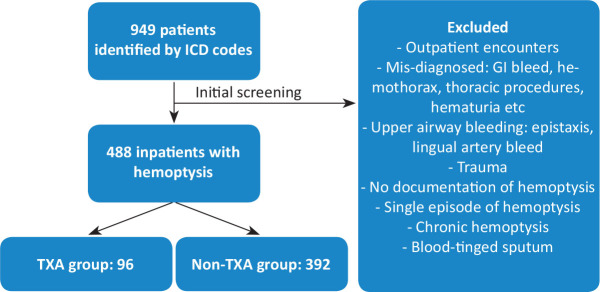
Flowchart of patient eligibility screening

We identified a cohort of individuals hospitalized at Indiana University Health University Hospital, Indiana University Health Methodist Hospital, or the Sidney and Lois Eskenazi Hospital during the period of interest. This was performed through the Regenstreif Institute in Indianapolis, IN, which serves as a data warehouse for Indiana University Health and Eskenazi Health. The Regenstrief Data Services identified individuals hospitalized with suspected hemoptysis using International Classification of Diseases Tenth revision codes (R04, R04.0, R04.1, R04.2, R04.8, R04.81, R04.89, R04.9). We limited our final analysis to codes R04.2, R04.89 and R04.9 and extracted prespecified clinical and demographic data from these charts. Outpatients were not included (Figure 1).

The charts of all suspected cases of hemoptysis were reviewed. Hemoptysis was defined as the expectoration of blood, at least two times over 48 hours. Blood-streaked sputum was not included. Both massive (expectorant blood >100 ml/hour, >250 ml per 24 hours and/or hemodynamically unstable) and non-massive hemoptysis were included. We included patients with identifiable and unidentifiable sites of bleeding. For the cohort of individuals who received TXA via nebulizer, the administered dose was 500 mg three times a day for 3 days at all three institutions per institutional order sets. Standard small volume nebulizer cups from various suppliers (Table 2) were used to deliver nebulized TXA, fiberoptic bronchoscopy was not used as a method of drug administration. The decision to use TXA, including use beyond 3 days, was at provider discretion. Timing of TXA initiation did not interfere with clinical care and decision making such as ICU admission, intubation, or invasive management.

**Table 2.** TXA sources

**Code**	**Source**
81284-0611-10	PROVEPHARM INC GWSA
67457-0197-10	MYLAN INSTITUTIONAL LLC
39822-1000-01	XGEN PHARM DJB INC
55150-0188-10	EUGIA US LLC
83634-0401-10	AVENACY INC GWSA
72485-0510-10	ARMAS PHARMACEUTICALS INC
00013-1114-21	PFIZER USPG
60505-6169-01	APOTEX CORPORATION
67850-0041-10	AVET PHARMACEUTICALS INC
61990-0611-02	PROVEPHARM INC GWSA
00517-0960-10	AMERICAN REGENT INC
47781-0601-91	ALMAJECT INC
72611-0760-10	ALMAJECT INC
70860-0400-10	ATHENEX PHARMACEUTICAL DIVISION
70860-0407-10	ATHENEX PHARMACEUTICAL DIVISION

The participating institutions are all part of Indiana University School of Medicine, but faculty physicians typically attend at only a single institution. Treatment algorithms for hemoptysis as well as local practice culture were shared among all three institutions. The identified patients were not part of any study involving TXA.

To be included in the analysis, patients had to be ≥ 18 years old, hospitalized at one of the three institutions listed above with a primary or secondary diagnosis of hemoptysis. We excluded patients who had a documented allergy to TXA, were pregnant at the time of admission or had contraindications to the use of TXA (venous or arterial thromboembolism, hypercoagulable state, or active thromboembolic disease).

Demographic data and clinical characteristics (age, gender, comorbid conditions – particularly underlying lung disease, use of anticoagulant (AC), or antiplatelet (AP) agents, smoking status, etiology of hemoptysis, and final diagnosis), severity variables (labs on admission and APACHE II scores), and outcome variables (hospital/ICU length of stay (LOS), need for mechanical ventilation (MV) or bronchoscopy, angiographic embolization, and mortality) were manually extracted from the medical record for analysis. All extracted demographic data was obtained at the time of admission before TXA administration.

The primary exposure variable was the use of TXA, with a secondary analysis limited to only those individuals who required ICU admission. The primary outcome variables were hospital and ICU length of stay. Secondary outcomes included mortality, adverse drug effects, and the need for invasive procedures including bronchoscopy or interventional radiology intervention. Differences between the TXA and non-TXA groups were determined using t-test for continuous variables and chi-square or fishers’ exact test for categorical variables.

An independent statistician performed the final analysis. Patient gender, smoking status, platelet count, AC/AP use, international normalized ratio (INR), category, and diagnosis variables were used to generate a propensity score for each patient [5]. A logistic regression model with nebulized TXA as a predictor and death as an outcome controlling for the propensity score was used to determine the association between the outcome and predictor. Propensity score analysis was used as the patients in our study were non-randomized. The analysis was done using SAS 9.4 (Cary, NC). A 5% significance level was used for all the tests.

## Results

Initial data extraction identified 949 individuals in the cohort (Figure 1). After the initial screening, 488 individuals were determined to meet all inclusion criteria for analysis. Ninety-six patients received nebulized TXA, and 392 did not.

There was no significant difference in gender distribution, average age, underlying coagulopathy, or underlying conditions between the two groups (Table 2). The mean age was 56.8 years, with a male predominance. There were slightly more smokers (p = 0.04) in the non-TXA group.

**Table 2.** Demographic information of all patients

**Demographic information**	**All patients (N = 488)**	**TXA (N = 96)**	**No TXA (N = 392)**	**p-value**
Gender:				
Female	177 (36.3%)	41 (42.7%)	136 (34.7%)	0.14
Male	311 (63.7%)	55 (57.3%)	256 (65.3%)	
Mean Age (SD)	56.8 (16.7)	58.0 (15.5)	56.6 (17.0)	0.44
Smokers	110 (22.5%)	14 (14.6%)	96 (24.5%)	0.04
AC/AP use	313 (64.1%)	75 (78.1%)	238 (60.7%)	<0.01
INR >= 1.5	108 (22.1%)	23 (24%)	85 (21.7%)	0.63
Median Platelet count (q1-q3)	196 (84–277)	197 (84–282)	192.5 (78–258.5)	0.36

**Underlying condition**				
Liver disease	42 (8.6%)	6 (6.3%)	36 (9.2%)	0.19
Bronchiectasis	45 (9.2%)	7 (7.3%)	38 (9.7%)	
Lung cancer/metastasis	58 (11.9%)	9 (9.4%)	49 (12.5%)	
Head/Neck cancer	16 (3.3%)	7 (7.3%)	9 (2.3%)	
Hematological cancer	30 (6.2%)	7 (7.3%)	23 (5.9%)	
Other cancer	31 (6.3%)	8 (8.3%)	23 (5.9%)	
None of the above	266 (54.5%)	52 (54.2%)	214 (54.6%)	

50% of patients required ICU admission (Table 3). Patients in the TXA group were more likely to need ICU admission, require mechanical ventilation and bronchoscopy (p<0.01) (Table 3). These patients also had a longer hospital stay and a higher mortality rate. We saw a 38.9% mortality in the entire cohort of patients. There was no significant difference between the groups in terms of needing intervention by radiology. Alveolar bleeding, defined as diffuse alveolar hemorrhage of any cause, was the most common underlying diagnosis in the TXA group (Table 3). This was the only statistically significant difference in the final diagnosis between the two groups. Infection was the most common etiology in the non-TXA group.

**Table 3.** Outcomes of all patients

**Outcomes**	**All patients (N = 488)**	**TXA (N = 96)**	**No TXA (N = 392)**	**p-value**
ICU admission	[N=224] 50%	[N=72] 75%	[N=151] 38.5%	<0.01
Average ICU LOS	10.5 (16.5)	16.9 (17.6)	7.3(14.9)	<0.01
Mechanical ventilation	155 (31.8%)	64 (66.7%)	91 (23.2%)	<0.01
Bronchoscopy	245 (50.2%)	76 (79.2%)	169 (43.1%)	<0.01
IR	29 (5.9%)	8 (8.3%)	21 (5.4%)	0.27
Average Hospital LOS	12.8 (17.1)	24.4 (27.8)	10 (11.6)	<0.01
Expired/Hospice	194 (39.8%)	56 (59%)	138 (35.2%)	<0.01

**Diagnosis**				
Alveolar bleeding	82 (16.8%)	29 (30.2%)	53 (13.5%)	<0.01
Infection	161 (33%)	26 (27.1%)	135 (34.4%)	0.17
Pulmonary edema	33 (6.8%)	3 (3.1%)	30 (7.6%)	0.11
Malignancy	73 (15%)	11 (11.5%)	62 (15.8%)	0.28
Coagulopathy	15 (3.1%)	4 (4.2%)	11 (2.8%)	0.51
Tracheal/bronchial bleeding	91 (18.7%)	16 (16.7%)	75 (19.1%)	0.58
Vascular abnormality	21 (4.3%)	3 (3.1%)	18 (4.6%)	0.78
Undetermined	12 (2.5%)	4 (4.2%)	8 (2.1%)	0.26

A subgroup analysis of patients admitted to the ICU was then performed (Table 4). The two groups were not statistically significantly different in terms of gender distribution, average age, smoking status, use of AC/AP, underlying coagulopathy, or underlying conditions. Once again, ICU LOS, hospital LOS, need for MV, need for bronchoscopy, and mortality were higher in the group receiving TXA (p<0.01) (Table 5). There was no statistically significant difference between the two groups in terms of the final diagnosis.

**Table 4.** Demographic information of all ICU patients

**Demographic information**	**ALL ICU (N = 223)**	**ICU + TXA (N = 72)**	**ICU + No TXA (N = 151)**	**p-value**
Gender:				
Female	84 (37.7%)	31 (43.1%)	53 (35.1%)	0.25
Male	139 (62.3%)	41 (56.9%)	98 (64.9%)	
Average Age	58.4 (16.3)	58.1 (16.1)	58.6 (16.4)	0.85
Smokers	46 (20.6%)	12 (16.7%)	34 (22.5%)	0.31
AC/AP use	169 (75.8%)	59 (81.9%)	110 (72.9%)	0.14
INR >= 1.5	55 (24.7%)	17 (23.6%)	38 (25.2%)	0.80
Median Platelet count (q1-q3)	178.5 (84–262)	165.5 (78–246)	181 (85–268)	0.64

**Underlying condition**				
Liver disease	26 (11.7%)	5 (6.9%)	21 (13.9%)	0.64
Bronchiectasis	8 (3.6%)	3 (4.2%)	5 (3.3%)	
Lung cancer/metastasis	22 (9.9%)	5 (6.9%)	17 (11.3%)	
Head/Neck cancer	10 (4.5%)	4 (5.6%)	6 (4%)	
Hematological cancer	11 (4.9%)	3 (4.2%)	8 (5.3%)	
Other cancer	18 (8.1%)	6 (8.3%)	12 (8%)	
None of the above	128 (57.4%)	46 (63.9%)	82 (54.3%)	

To determine if severity of illness was contributing to the use of TXA, we did a subgroup analysis of the ICU patients using APACHE II scores (Table 5), calculated at the time of admission. However, the median APACHE II scores between the two groups were not significantly different.

**Table 5.** Outcomes of ICU patients

**Outcomes**	**ALL ICU (N = 223)**	**ICU + TXA (N = 72)**	**ICU + No TXA (N = 151)**	**p-value**
Median APACHE II scores	17.6 (8.5)	18.7 (9.1)	17.1 (8.1)	0.17
MV	147 (65.9%)	59 (81.9%)	88 (58.3%)	<0.01
Bronchoscopy	155 (69.5%)	61 (84.7%)	94 (62.3%)	<0.01
IR	17 (7.6%)	6 (8.3%)	11 (7.3%)	0.78
Average ICU LOS	10.3 (16.4)	16.6 (17.5)	7.3 (15)	<0.01
Average Hospital LOS	16.5 (18.3)	23.7 (19.9)	13.1 (16.5)	<0.01
Expired/Hospice	113 (50.9%)	44 (62%)	69 (45.7%)	0.02

**Diagnosis**				
Alveolar bleeding	60 (26.9%)	25 (34.7%)	35 (23.2%)	0.07
Infection	68 (30.5%)	20 (27.8%)	48 (31.8%)	0.54
Pulmonary edema	12 (5.4%)	1 (1.4%)	11 (7.3%)	0.11
Malignancy	25 (11.2%)	6 (8.3%)	19 (12.6%)	0.50
Coagulopathy	8 (3.6%)	1 (1.4%)	7 (4.6%)	0.44
Tracheal/bronchial bleeding	33 (14.8%)	14 (19.4%)	19 (12.6%)	0.18
Vascular abnormality	10 (4.5%)	2 (2.8%)	8 (5.3%)	0.51
Undetermined	7 (3.1%)	3 (4.2%)	4 (2.6%)	0.68

The propensity analysis for death showed an increased odds ratio (OR) of 2.51 in the patients that received nebulized TXA (Table 6).

**Table 6.** Propensity score analysis for death

**Variable**	**Odds Ratio**	**Confidence Intervals**	**p-value**
TXA Use:			
Yes	2.51	1.56–4.02	<0.01
No	Reference		

In terms of adverse effects, there were 3 superficial clots, 4 deep venous thromboembolisms, 1 arterial thromboembolism, and 8 acute strokes reported in the group of ICU patients that received TXA. There was also one case of breakthrough seizures in a patient with a known seizure disorder. These adverse effects were not seen in the ICU non-TXA group. Death was primarily from respiratory failure and not as a result of adverse events.

## Discussion

This is the largest sample size comparing the clinical outcomes of hospitalized patients with both massive and non-massive hemoptysis receiving nebulized TXA [1,2,3,4,5]. 50% of the patients hospitalized with hemoptysis required ICU admission with the entire cohort of patients having a 39.8% mortality [Table II]. To the best of our knowledge, our study is the first study to show worse clinical outcomes with using nebulized TXA for hemoptysis.

In terms of our primary outcome, we found that patients who received TXA were more likely to be admitted to the ICU and they had longer ICU and hospital length of stays [Tables II & IV]. Patients who got TXA and were admitted to the ICU also had a statistically significantly higher mortality rate [Table IV]. The finding of increased mortality is independent of disease severity as measured by APACHE II scores at the time of admission in the subgroup of individuals hospitalized in the ICU [Table IV]. It is possible that there is some selection bias even though the two groups did not appear different in terms of illness severity. Physicians may have chosen to give TXA to the patients that appeared to have more severe disease introducing selection bias. This likely also contributed to the numerical difference between the TXA and non-TXA groups.

The reason behind the increased mortality among the individuals who received TXA is unclear. We suspect that known side effects of TXA, including clot formation and bronchospasm [2, 4], may be contributing factors as respiratory failure was the primary reason for death. A study in mice by Sperzel et al. showed a concerning dose dependent increase in thrombosis both in vivo and in vitro with TXA use [8]. Thus, we also suspect that increased V/Q mismatch and dead space ventilation may be worsening outcomes.

Prior studies in the use of TXA for hemoptysis have yielded conflicting results. To date, there have been only two small (sample size ranging 40–100), single center, randomized control trials of hospitalized patients showing the effectiveness of nebulized TXA in controlling bleeding [1, 2]. A systematic review and meta-analysis suggested decreased short-term mortality with the use of intravenous TXA, but this was not statistically significant [5]. On the other hand, a nationwide Japanese study showed a statistically significant decrease in hospital mortality with use of intravenous tranexamic acid for hemoptysis [6]. In addition, Wang et al. reported a 2% reduction in long-term mortality with nebulized tranexamic acid [1].

In the meta-analysis by Liang et al. examining the various formulations of TXA for hemoptysis; there was an association between smoking, malignancy, pulmonary infections, and AC use with increased mortality [5]. We therefore elected to use these factors in our propensity score analysis. However, despite more patients with these factors being in the non-TXA group, the group receiving nebulized TXA still had a higher mortality rate in our study [Tables II and IV].

Unlike other studies, we found that the patients receiving TXA were more likely to receive bronchoscopy to control or locate bleeding [1–2, 5]. The documented rationale for most of these procedures was to localize the bleeding site. We did not find a significant difference in the need for bronchial artery embolization, as reported by Gopinath and Liang-Fu [2, 5].

Given the growing popularity in the use of TXA for hemoptysis, we suspect that more patient cases of adverse effects like that published by Hardin et al. will become apparent in the future. At this time, we still do not have a lot of published data in the use of TXA for hemoptysis.

## Conclusions

In summary, we found that nebulized TXA was associated with increased average length of ICU and hospital length of stay, need for mechanical ventilation, need for bronchoscopy, and higher in-hospital mortality. This association remained true even after a propensity analysis adjusting for severity of illness. The design of our study limits the ability to determine cause and effect and is open to potential bias. Larger randomized trials (with significantly more patients) and more research is needed to exclude other confounding factors and confirm an association between nebulized TXA and worse outcomes.

## References

[1] Wand O, Guber E, Guber A. (2018). Inhaled tranexamic acid for hemoptysis treatment: a randomized controlled trial. Chest.

[2] Gopinath B, Mishra PR, Aggarwal P (2023). Nebulized vs IV Tranexamic Acid for Hemoptysis: A Pilot Randomized Controlled Trial. Chest.

[3] Solomonov A, Fruchter O, Zuckerman T (2009). Pulmonary hemorrhage: A novel mode of therapy. Respir Med.

[4] Alabdrabalnabi F, Alshahrani M, Ismail N (2020). Nebulized tranexamic acid for recurring hemoptysis in critically ill patients: case series. J Emerg Med.

[5] Chen LF, Wang TC, Lin TY (2021). Does tranexamic acid reduce risk of mortality on patients with hemoptysis? A protocol for systematic review and meta-analysis. Med.

[6] Myles PS, Smith JA, Forbes A (2017). Tranexamic acid in patients undergoing coronary-artery surgery. N Engl Journal of Med.

[7] Hardin J, Seltzer J, Moriguchi R (2024). Tranexamic Acid Neurotoxicity After Nebulization and BAL. Chest.

[8] Sperzel M, Huetter J (2007). Evaluation of aprotinin and tranexamic acid in different in vitro and in vivo models of fibrinolysis, coagulation and thrombus formation. J Thromb Haemost.

